# Six-methoxyflavone suppresses CircPIAS1 biogenesis via targeting PTBP1 and, in combination with IFN-γ, promotes ferroptosis in melanoma

**DOI:** 10.3389/fphar.2025.1681890

**Published:** 2025-11-19

**Authors:** Yong-Qi Guo, Xi Wu, Xin Zang, Cheng-Yan Liu, Meng-Yue Wang, Cheng-Mei Xiao, Hui Hou, Li-Fang Zhang, Yuan-Zheng Xia, Ling-Yi Kong

**Affiliations:** 1 Basic Medical Research Innovation Center for Anti-Cancer Drugs, MOE and State Key Laboratory of Natural Medicines, China Pharmaceutical University, Nanjing, China; 2 Puhe Biopharma, Soochow, Jiangsu, China; 3 Guangxi Key Laboratory of Early Prevention and Treatment for Regional High Frequency Tumor and Key Laboratory of Early Prevention and Treatment for Regional High Frequency Tumor (Guangxi Medical University), Ministry of Education, Nanning, China

**Keywords:** circRNA, 6-methoxyflavone, ferroptosis, RNA binding protein, melanoma

## Abstract

**Background/Objectives:**

Melanoma remains a highly aggressive malignancy with limited therapeutic options targeting its underlying pathogenesis. CircPIAS1 (circbase ID: hsa_circ_0008378) and its encoded protein circPIAS1-108aa contribute to tumor progression by suppressing *STAT1* phosphorylation and immunogenic ferroptosis, yet specific pharmacological agents of directly targeting circPIAS1 are lacking. This study aimed to identify natural products that selectively inhibit circPIAS1 biogenesis, there-by exploring novel therapeutic strategies for melanoma.

**Methods:**

A library of 128 anticancer natural products was screened using qRT-PCR to identify metabolites selectively suppressing circPIAS1, with 6-methoxyflavone (6-MF) selected. CCK-8 assay determined 6-MF’s IC_50_ for inhibiting melanoma cell proliferation. RNA sequencing and western blot analyzed the *PI3K-AKT* pathway, *STAT1* phosphorylation, *SLC7A11/GPX4* signaling pathway, and circPIAS1-108aa. EdU and lipid ROS assays evaluated cell proliferation and oxidative response. RBPmap database identified PTBP1 as a key RNA-binding protein (RBP) in circPIAS1 biogenesis, validated via dose/time-dependent 6-MF treatments and siRNA experiments. A subcutaneous tumor model was used to evaluate the effects of 6-MF, a PD-1 inhibitor, and their combination.

**Results:**

6-MF was identified as a selective suppressor of circPIAS1 without affecting its host *PIAS1* gene. Mechanistically, 6-MF inhibited PTBP1 (critical for circPIAS1 biogenesis) in a dose- and time-dependent manner, reducing circPIAS1-108aa expression. It suppressed the *PI3K-AKT* pathway,and when combined with IFN-γ, it significantly enhanced *STAT1* phosphorylation, and downregulated *SLC7A11/GPX4*, increasing lipid ROS and promoting ferroptosis. *In vivo*, 6-MF combined with PD-1 inhibitor synergistically inhibited melanoma growth.

**Conclusion:**

6-MF targets *PTBP1* to inhibit circPIAS1 biogenesis and reduce circPIAS1-108aa.6-MF inhibits PI3K-AKT pathway; combined with IFN-γ, it enhances *STAT1* phosphorylation, inhibits *SLC7A11/GPX4* pathway, promoting melanoma ferroptosis. Its combination with PD-1 inhibitor enhances antitumor efficacy, providing a novel therapeutic strategy for melanoma treatment.

## 1 Introduction

Melanoma is a type of skin cancer that is known for its aggressiveness and increasing incidence, particularly in Europe (Conforti and Zalaudek, 2021; Robbins et al., 2015; Tinca et al., 2023). Although surgical resection is the primary treatment for early-stage melanoma, the 5-year survival rate for patients with advanced melanoma (stage III/IV) is only 15%–20%. In recent years, targeted therapy and immunotherapy have become first-line treatments. PD-1 inhibitors, such as nivolumab and pembrolizumab, has demonstrated notable efficacy and prolonged responses when utilized as postoperative adjuvant therapy. PD-1, a critical immune checkpoint protein, is predominantly expressed on the surface of activated T cells. When PD-1 binds to its ligand, PD-L1, it inhibits the activation and proliferation of T cells, thereby allowing melanoma cells to evade immune detection and suppression. However, it should be noted that not all patients benefit from immune checkpoint blockade (ICB) therapy, and the response of different patients to ICB varies widely. Some patients achieve long-term disease remission, while others experience disease progression (Gellrich et al., 2020; Lee et al., 2016). The mechanism is not fully understood, especially the role of non-coding RNA (such as circular RNA or circRNA) in regulating immune responses or treatment sensitivity.

Interferon-γ (IFN-γ) is a protein encoded by the *IFNG* gene and consists of two antiparallel linked polypeptide chains. In the context of melanoma, IFN-γ exhibits a dual role, functioning as both a pivotal element in immune activation and a catalyst for therapeutic resistance through cancer-promoting pathways, such as *FGF2/Wnt* (Jorgovanovic et al., 2020; Li et al., 2023). The 6-MF employed in this study is a naturally occurring flavonoid metabolite that has been documented to possess antibacterial, antioxidant, anticancer, anti-tumor, and immunomodulatory properties (Huang, 2025). Choi found that Aurantica induced reactive oxygen species (ROS) production, regulated apoptosis-related proteins, and induced apoptosis in melanoma cells (Lee et al., 2019). Fisetin, a dietary flavonoid, reduced MITF levels by interfering with the Wnt/β-catenin pathway, inhibited the growth of human melanoma cells, and induced G1-phase blockade (Syed et al., 2011). However, it needs to be clarified whether 6-MF affects the antitumor effects of IFN-γ by regulating circRNA.

CircRNA is a non-coding molecule that is produced from exons and/or introns of a gene by means of back-splicing. CircRNAs are expressed in various tissues and cell types in a time-regulated manner (Liang et al., 2021; Mahmoudi and Cairns, 2023). CircRNA can act as a microRNA (miRNA) sponge, participate in transcriptional regulation, splicing interference, and other functions (Liu and Chen, 2022; Zhou et al., 2020; Tong et al., 2022; Wang et al., 2019; Li et al., 2021). Furthermore, circRNAs have been shown to play a role in melanoma proliferation, drug resistance, and other processes. For example, circ_0020710, highly expressed in melanoma, is associated with poor prognosis, and recruits Tregs and MDSCs to form an immunosuppressive microenvironment through a ceRNA mechanism (competitive adsorption of miR-370-3p to deregulate CXCL12). Knocking down circ_0020710 and combining it with a PD-1 inhibitor shrink the tumor and reverse the immunosuppression, providing a potential target for combination therapy (Wei et al., 2020). While the role of circPIAS1 (circbase ID: hsa_circ_0008378) in melanoma has been the subject of study, the drugs that act on circPIAS1 and their mechanism of action have not been investigated.

The biogenesis of circRNA involves a complex process that comprising reverse splicing, cis-acting elements, such as the side-upside-down Alu element, and trans-acting elements, including RNA-binding proteins (RBPs). Of these, RBPs play a pivotal role in reverse splicing, as RBPs are capable of recognizing and binding to two introns on pre-mRNA, thereby facilitating the biogenesis of circRNA. Currently, the most extensively studied RBPs include QKI, FUS, and MBNL1 (Conn et al., 2024; Li et al., 2020; Pisignano et al., 2023; Zhao et al., 2021). For instance, the QKI has been shown to be responsible for the biogenesis of circPRMT10 by binding to the flanking intron of circPRMT10 (Xiao et al., 2025). In certain tumor cells, the RBP protein hnRNPA1 has been observed to bind to the reverse splicing enhancer region of specific gene introns. This binding event has been shown to impede the binding of proteins that promote reverse splicing, consequently leading to a decrease in circMEF2D production and a relative increase in linear mRNA production (Zhang et al., 2022). PTBP1, an RBP, functions as a conserved splicing regulator. It modulates diverse target genes through selective splicing, a process that is tissue-specific and context-dependent. PTBP1 plays a pivotal role in regulating cell fate and disease processes (Liu et al., 2023; Taniguchi et al., 2021). PTBP1 functions as an RBP and may play a pivotal role in circRNA biogenesis. However, the association between PTBP1 and circRNA in melanoma remains to be elucidated.

The extant literature has not revealed a correlation between 6-MF and circRNA. This study, however, is the first to identify the specific regulation of the *PIAS1* circRNA (circPIAS1) by 6-MF (without affecting its host gene *PIAS1*). In this study, the CCK-8 assay was used to detect the effect of circPIAS1 on melanoma cell proliferation and the IC_50_ value. RNA sequencing and Western blot were used to clarify the signaling pathway and protein abundance associated with circPIAS1 function. Cell proliferation and the oxidative stress response were analyzed using an EdU assay and a lipid ROS assay. The association between the RNA-binding protein PTBP1 and circPIAS1 biogenesis was explored using the RBPmap database and cell experiments. Meanwhile, the *in vivo* efficacy of 6-MF in combination with PD-1 inhibitors was validated using a melanoma subcutaneous xenograft model. Our findings suggest that the natural metabolite 6-MF can inhibit PTBP1 expression in melanoma cells in a dose- and time-dependent manner, thereby reducing circPIAS1 biogenesis and circPIAS1-108aa protein expression. 6-MF interferes with the *PI3K-AKT* signaling pathway. When combined with IFN-γ, it significantly enhances *STAT1* phosphorylation levels and inhibits the *SLC7A11/GPX4* axis, thereby promoting ferroptosis in melanoma cells. Furthermore, the combination of 6-MF and PD-1 inhibitors effectively enhances the efficacy of immune checkpoint blockade therapy and inhibits tumor growth. These findings reveal a novel mechanism by which 6-MF regulates circPIAS1, providing a potential targeting strategy for melanoma treatment.

## 2 Materials and methods

### 2.1 Cell lines and cell culture

The human A375 cells and mouse B16-F10 cells utilized in this study were obtained from the Shanghai Cell Bank of the Chinese Academy of Sciences. The cells were cultivated in Dulbecco’s Modified Eagle Medium (DMEM) medium, with the addition of 10% fetal bovine serum (FBS), and were maintained at a temperature of 37 °C in an atmosphere containing 5% CO_2_.

### 2.2 Cell viability assay

Human A375 cells and murine B16-F10 melanoma cells were cultivated in 96-well plates and exposed to 6-MF at concentrations of 0, 5, 10, 20, 40, 60, and 80 μM for a duration of 48 h. Each treatment group comprised three replicate wells. Subsequent to the treatment period, 10 μL of CCK-8 solution (Run Yan Bio, CT01A) was added to each well, and the optical density (OD) at 450 nm was measured to calculate the drug’s inhibition rate on the cells.

### 2.3 EdU cell proliferation assay

Human A375 and murine B16-F10 cells were seeded in 96-well plates (1 × 10^4^ cells/well) and cultured overnight. Cells were treated with 6-MF, IFN-γ or the combination treatment of both for 48 h, followed by incubation with 1 × EdU (Beyotime, C0071L) solution for 6 h. After fixation and permeabilization (0.1% Triton X-100), 50 μL reaction solution was added and incubated for 30 min (dark, room temperature). Nuclei were counterstained with DAPI before imaging.

### 2.4 Cell protein extraction

Human A375 cells were cultivated and subsequently harvested. The cell pellet was then lysed using WB/IP cell lysis buffer (Beyotime, P0013) and subjected to sonication. The lysate was subsequently incubated on ice and then subjected to centrifugation. The resulting pellet was then collected for further analysis.

### 2.5 RNA sequencing

The human A375 cells were divided into four experimental groups: control group, 6-MF treatment group, IFN-γ treatment group, and 6-MF combined with IFN-γ treatment group. Following the administration of drugs, cell precipitates were collected by centrifugation and stored at −80 °C for subsequent RNA sequencing analysis. The sequencing process was carried out using computational platforms.

### 2.6 Western blot experiment

Following the extraction of the proteins, their quantification was conducted using the BCA kit (Beyotime, P0013). A total of 30 μg of protein was added to each sample. The samples were then electrophoresed on 10% SDS-PAGE. Thereafter, they were transferred to a PVDF membrane. The PVDF membrane was blocked at room temperature using the TBST solution of 5% skimmed milk. The samples were then incubated overnight using the specific antibodies. They were subsequently incubated with the secondary antibodies. The PVDF membrane was exposed using the ECL (EpiZyme SQ201) solution, and details of the antibodies used are given in Supplementary Table S2.

### 2.7 Quantitative real-time PCR (qRT-PCR)

The RNA was extracted using RNA extraction kits (SHANGHAI YISHAN, ES-R001 and ES-RN002plus) and reverse transcribed to cDNA (HiScript® II Reverse Transcriptase, Vazyme, R211-01). The resulting cDNA was then combined with primers and SYBR Green (Taq Pro Universal SYBR qPCR Premix, Vazyme, Q311-02) in specific ratios for qRT-PCR. The relative RNA content was subsequently determined using the formula 2^−ΔΔCT^. and the specific primers used in this study are listed in Supplementary Table S1.

### 2.8 Lipid ROS assay

Subsequent to treatment, the original medium was discarded and replaced with fresh medium containing 5 μmol/L C11-BODIPY 581/591 (Invitrogen™, C10445). The cells were then incubated at room temperature in the dark for a period of 1 h. Subsequently, Hoechst staining solution was added to stain the nuclei, and cell images were captured after staining. The shift in the fluorescence emission peak of C11-BODIPY 581/591 from 590 nm (red) to 510 nm (green) is indicative of lipid hydroperoxide-mediated oxidation of the cell membrane, leading to lipid peroxidation damage. The extent of oxidative damage to the cell membrane was then quantified by determining the ratio of green to red fluorescence intensity.

### 2.9 Transfection experiments

Small interfering RNA (siRNA) for transient transfection was obtained from RiboBio Co., Ltd. (Guangzhou, China). SiRNA transfection was performed using Lipofectamine™ 3000 (Thermo Fisher Scientific, L3000015) and P3000™ reagent (Thermo Fisher Scientific, L3000015) in Opti-MEM™ medium. After transfection, cells were collected, and proteins were extracted for subsequent western blot analysis. The siRNA sequences used in this study are listed in Supplementary Table S3.

### 2.10 Animal experiments

To establish a melanoma cancer model, 6–8-week-old C57/6J female mice (SPF) were utilized for subcutaneous injection of 2 × 10^5^ melanoma B16-F10 cells. When the tumor reached 50 mm^3^, the mice were divided into four groups (n = 6), including 6-MF, PD-1 inhibitor, 6-MF and PD-1 combination group (IgG was used as a negative control). The pharmaceutical agents were administered at an interval of 3 days, The PD-1 inhibitor and the IgG negative control were administered at a concentration of 2 mg/kg. The dosage of 6-MF was 60 mg/kg, and the intraperitoneal injection was administered once every 3 days for a total of five times. Tumor tissues were collected from each group on the 14th day for further analysis. The animal experiment plan was reviewed and approved by the Institutional Animal Care and Use Committee (IACUC) of the Experimental Animal Center of China Pharmaceutical University (2024-11-107).

### 2.11 Ethical approval for animal experiments

The animal experimental protocol underwent examination and approval by the Institutional Animal Care and Use Committee (IACUC) of the China Pharmaceutical University Experimental Animal Center (2024-11-107).

### 2.12 Statistical analysis

*In vitro* experiments were independently repeated at least three times. Statistical significance was assessed using GraphPad Prism 10.5.0, with group differences analyzed by Student’s t-test and one-way analysis of variance (ANOVA). Venn diagram was generated with Origin2021. The resulting data are presented as “mean ± standard deviation (SD)”. Statistical significance is indicated as follows: ns (not significant), ^*^ *P* < 0.05 (significant), ^**^ *P* < 0.01 (highly significant).

## 3 Results

### 3.1 The natural product 6-MF shows the inhibitory effect on the proliferation of melanoma cells and circPIAS1

**FIGURE 1:**
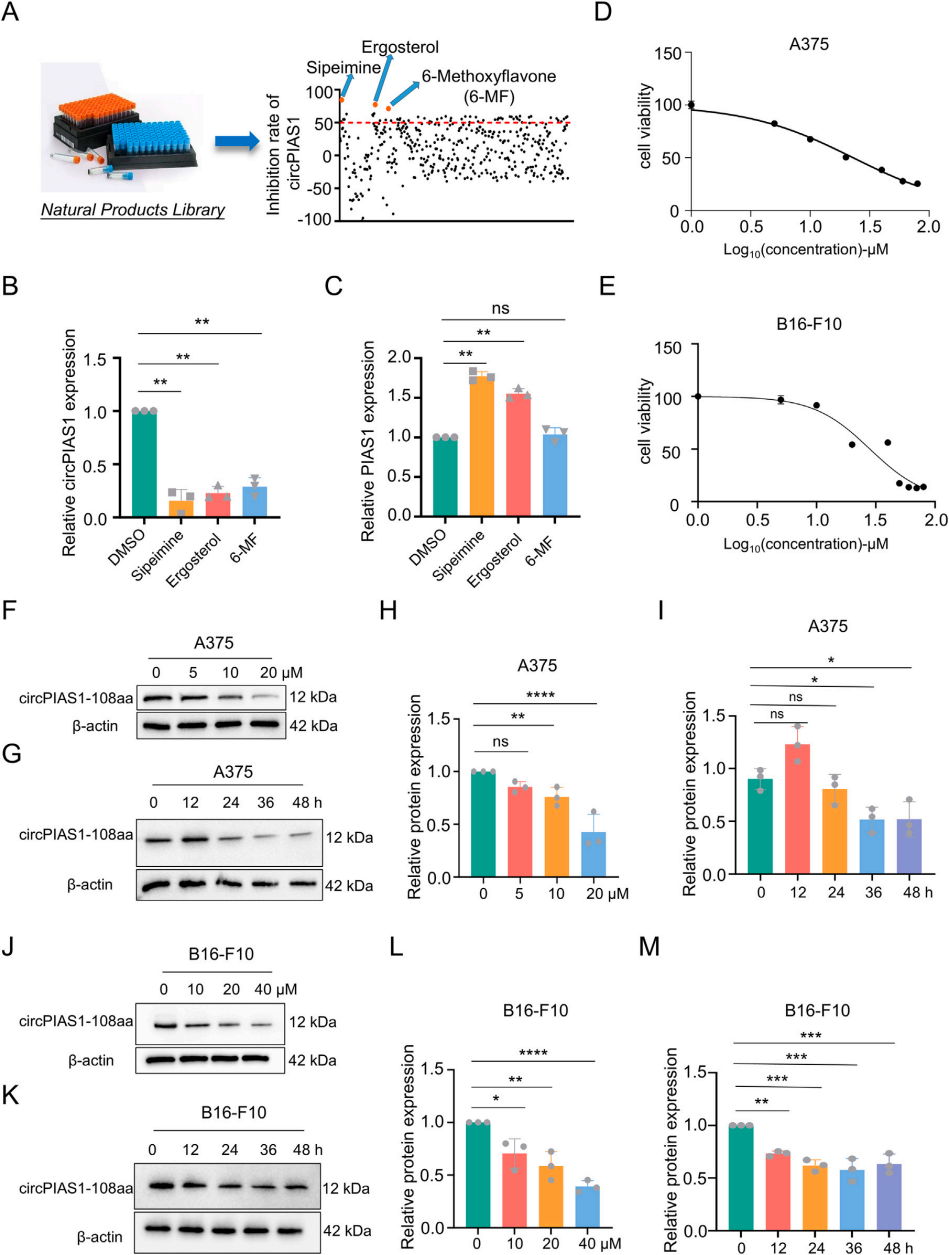
The natural product 6-MF shows the inhibitory effect on the proliferation of melanoma cells and circPIAS1. **(A,B)** Out of 128 drug candidates, three drugs that can reduce circPIAS1 were identified using the qRT-PCR method (n = 3 independent experiments). **(C)** The qRT-PCR method identified 6-MF as a metabolite that can inhibit circPIAS1 without affecting its host genes. **(D,E)** CCK-8 assay on A375 and B16-F10 cells under 5 μM–100 μM 6-MF conditions. Five thousands cells were cultured in 96-well plates for 48 h, and then absorbance at 450 nm was determined to obtain an inhibitory effector dose-effect curve with IC_50_ values. **(F)** Western blot analysis of circPIAS1-108aa in A375 cells after 48 h treated with different concentrations of 6-MF. β-ACTIN was used as an internal control (n = 3 independent experiments). **(G)** Western blot analysis of circPIAS1-108aa in A375 cells treated with 6-MF at a concentration of 20 μM at different times. β-ACTIN was used as an internal control (n = 3 independent experiments). **(H,I)** The relative protein levels of circPIAS1-108aa were analyzed in A375 cells following the addition of 6-MF at various concentrations and time points (n = 3 independent experiments). **(J)** Western blot analysis of circPIAS1-108aa in B16-F10 cells after 48 h treated with different concentrations of 6-MF. β-ACTIN was used as an internal control (n = 3 independent experiments). **(K)** Western blot analysis of circPIAS1-108aa in B16-F10 cells treated with 6-MF at a concentration of 20 μM at different times. β-ACTIN was used as an internal control (n = 3 independent experiments). **(L,M)** Relative protein levels of circPIAS1-108aa were assessed by western blot analysis in B16-F10 cells after the addition of 6-MF at different concentrations and time points (n = 3 independent experiments). Data represent three experiments and are expressed as mean ± SD: ns (not significant); ^*^ *P* < 0.05; ^**^ *P* < 0.01 (significant). Data were analyzed by One-way ANOVA **(B,C,H,I,L,M)** in GraphPad Prism 10.5.0.

In previous studies, the mechanism by which an oncogenic PIAS1 variant (circPIAS1-108aa) encoded by circPIAS1 blocks immunogenic ferroptosis was investigated (Zang et al., 2024). Thus, a search was initiated for drugs that could reduce the production of circPIAS1-108aa from the source. Following the screening of 128 anti-cancer natural products from the metabolite library in our laboratory, three chemicals were identified (Figure 1A). Among them, 6-MF was found to be capable of downregulating circPIAS1, yet did not interfere with the levels of the host gene *PIAS1* (Figures 1B,C). The CCK-8 assay was conducted in order to ascertain the effect of 6-MF on the proliferation of melanoma cells and demonstrated that as the concentration of 6-MF increased, the cell viability was gradually inhibited (Figures 1D,E). The IC_50_ values of A375 and B16-F10 were 23.04 ± 1.91 μM and 28.39 ± 4.18 μM respectively A375 and B16-F10 cells were treated with 6-MF, and the results indicated that 6-MF significantly inhibited the expression of circPIAS1-108aa protein encoded by circPIAS1 in a concentration- and time-dependent manner (Figures 1F‐M ).

### 3.2 RNA-seq analysis and Western blot demonstrate that 6-MF suppresses PI3K-AKT signaling pathway in melanoma cells

**FIGURE 2:**
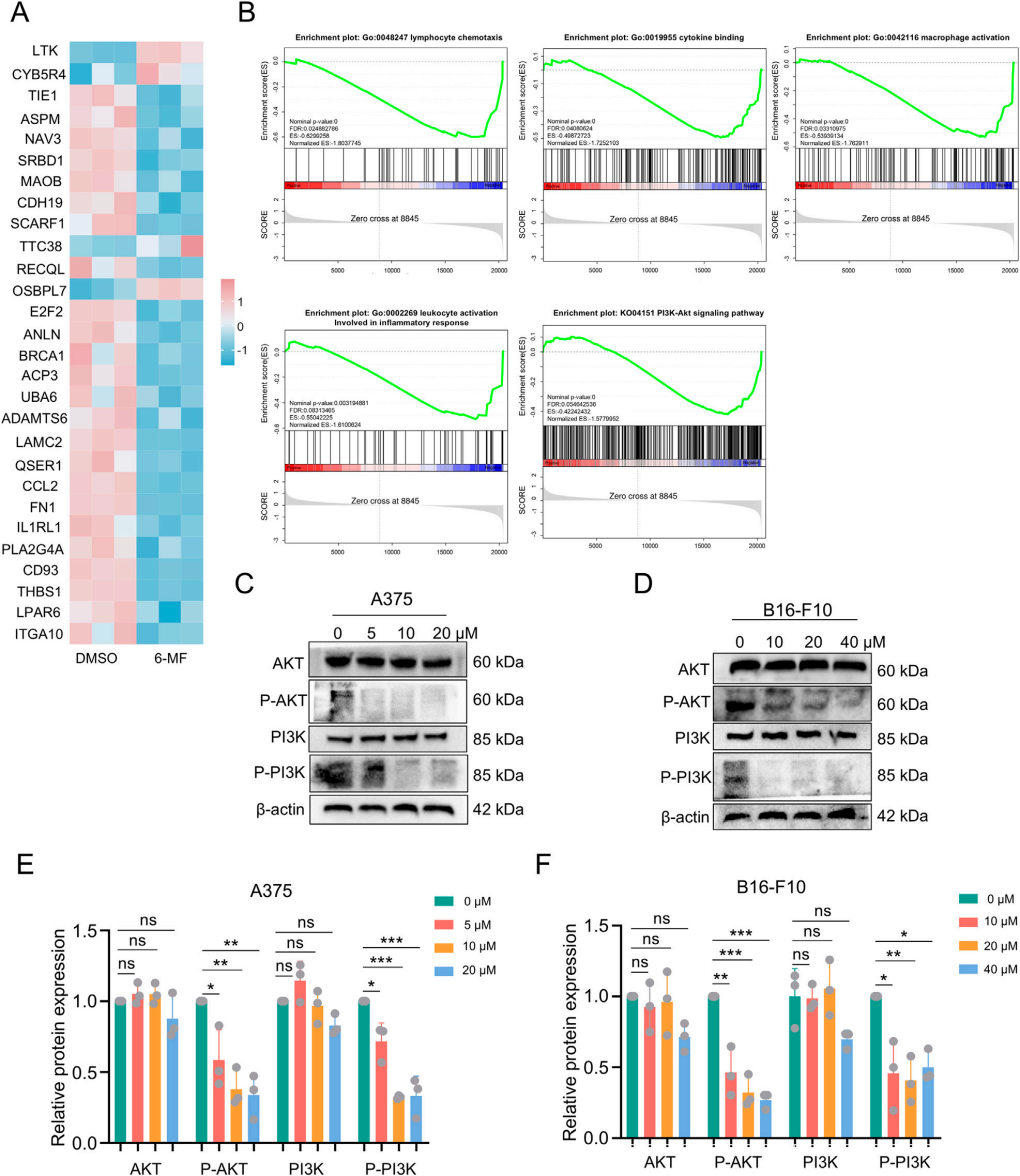
RNA-seq analysis and western blot demonstrate that 6-MF suppresses PI3K-AKT signaling pathway in melanoma cells. **(A)** RNA-seq screening identified potential genes with significant differences in four gene sets and one pathway in A375 cells. **(B)** RNA-Seq analysis comparing KEGG pathway analysis and GO analysis enrichment of A375 cells (negative control and 6-MF groups, the concentration of 6-MF was 20 μM) identified 4 gene sets and 1 pathway (n = 3 independent experiments). **(C,D)** Western blot analysis of the PI3K-AKT signaling pathway in A375 and B16-F10 cells after 48 h treated with different concentrations of 6-MF. β-ACTIN was used as an internal control (n = 3 independent experiments). **(E,F)** Relative protein levels of AKT, P-AKT, PI3K, and P-PI3K were analyzed and assessed after the addition of different concentrations of 6-MF (n = 3 independent experiments). Data are representative of three experiments and are expressed as mean ± SD. ns (not significant), ^*^ *P* < 0.05, and ^**^ *P* < 0.01 (significant). Data were analyzed by One-way ANOVA **(E,F)** in GraphPad Prism 10.5.0.

A375 cells were treated with 6-MF and then subjected to RNA sequencing to analyze transcriptional changes. The RNA-seq results (Figures 2A,B) illustrated that 6-MF significantly restrained lymphocyte chemotaxis, macrophage activation, leukocyte activation, cytokine binding and *PI3K-AKT* pathway, leading to the identification of 26 genes that exhibited significant correlations. As shown in the RNA-seq results above, the *PI3K-AKT* signaling pathway is a key regulator of the immune system, the lymphatic system, macrophages and inflammatory factors. It is also closely related to immune ICB therapy and ferroptosis (Fan et al., 2021; Zhu et al., 2025). In western blot experiments, 6-MF was found to exert a significant inhibitory effect on the levels of P-AKT and P-PI3K proteins without affecting the total PI3K and AKT protein in A375 and B16-F10 cells (Figures 2C–F).

### 3.3 The combination of 6-MF and IFN-γ inhibits the *SLC7A11/GPX4* axis via increasing *STAT1* phosphorylation and promotes ferroptosis in melanoma cells

**FIGURE 3:**
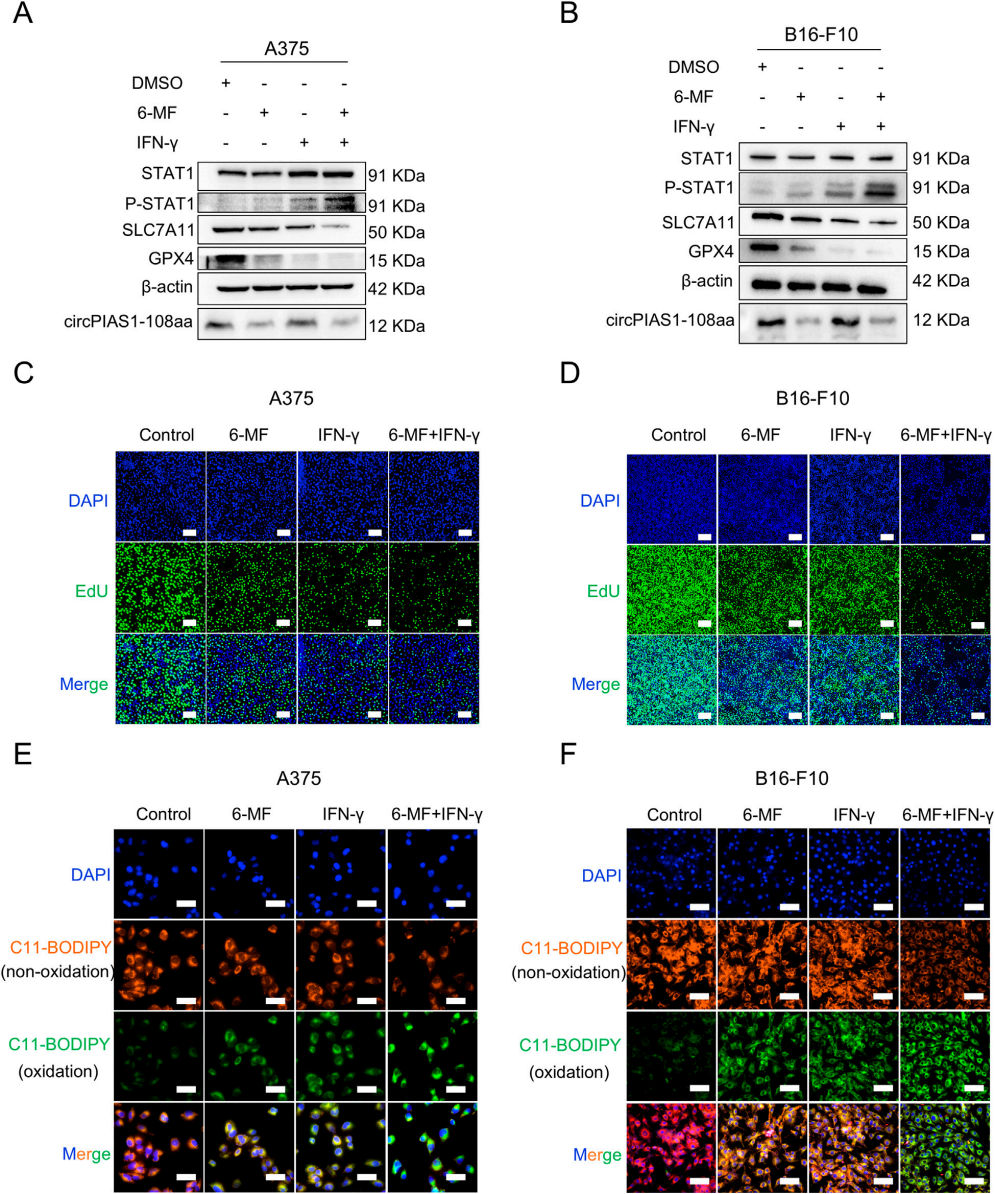
The combination of 6-MF and IFN-γ inhibits the SLC7A11/GPX4 axis via increasing STAT1 phosphorylation and promotes ferroptosis in melanoma cells. **(A,B)** Western blot experiments were performed using 6-MF and IFN-γ as well as their combination to treat A375 and B16-F10 cells for 48 h to detect the effects on the *P-STAT1/SLC7A11/GPX4* signaling pathway. β-ACTIN was used as an internal control (6-MF was used at a concentration of 20 μM, and IFN-γ at 2 ng/mL) (n = 3 independent experiments). **(C,D)** EdU assays were performed on A375 and B16-F10 cells, and 2 × 10^4^ cells were incubated in 96-well plates with 20 μM 6-MF, 2 ng/mL IFN-γ, and their combination for 48 h to verify the effect on cancer cell proliferation (scale bar: 100 μm) (n = 3 independent experiments). **(E,F)** ROS assays were performed on A375 and B16-F10 cells, 1 × 10^4^ cells were incubated in 96-well plates with 20 μM 6-MF, 2 ng/mL IFN-γ, and co-administration of 6-MF and IFN-γ for 48 h. The C11-BODIPY 581/591 probe was used to assess the effect on lipid peroxidation damage on the cell membrane surface (scale bar: 50 μm) (n = 3 independent experiments).

In previous studies, circPIAS1-108aa was found to reactivate the *SLC7A11/GPX4* pathway by inhibiting phosphor-STAT1 (P-STAT1), and IFN-γ was found to enhance STAT1 phosphorylation, thereby inhibiting downstream transduction of the *SLC7A11/GPX4* signaling, ultimately triggering immunogenic ferroptosis in cancer cells (Zang et al., 2024; Gao et al., 2025; Wu et al., 2023; Yi et al., 2020). The validation of this pathway was conducted using 6-MF and IFN-γ (Figures 3A,B; Supplementary Figure S1A,B). The treatment of 6-MF upregulated P-STAT1 levels. Besides, the stimulation with IFN-γ increased more P-STAT1 expression. Furthermore, the combination of 6-MF and IFN-γ led to the highest P-STAT1 levels. It was determined that the *SLC7A11/GPX4* axis was a key downstream target of *STAT1* (Yu et al., 2023). The protein levels of SLC7A11 and GPX4 were downregulated by 6-MF, IFN-γ or their combination in A375 and B16-F10 melanoma cells, indicating that ferroptosis might be induced in the presence of these therapeutic strategies. The EdU and lipid ROS assays confirmed that 6-MF decreased cell proliferation (Figures 3C,D; Supplementary Figure S1C) and increased oxidative reactions (Figures 3E,F; Supplementary Figure S1D), with IFN-γ having a stronger effect. The combination of 6-MF and IFN-γ exhibited the most potent inhibitory effect on proliferation and the strongest oxidative stress. The results of the present study demonstrate that the combination of 6-MF and IFN-γ can induce the ferroptosis of melanoma cells.

### 3.4 Six-MF blocks circPIAS1 biogenesis by reducing the expression levels of PTBP1

**FIGURE 4:**
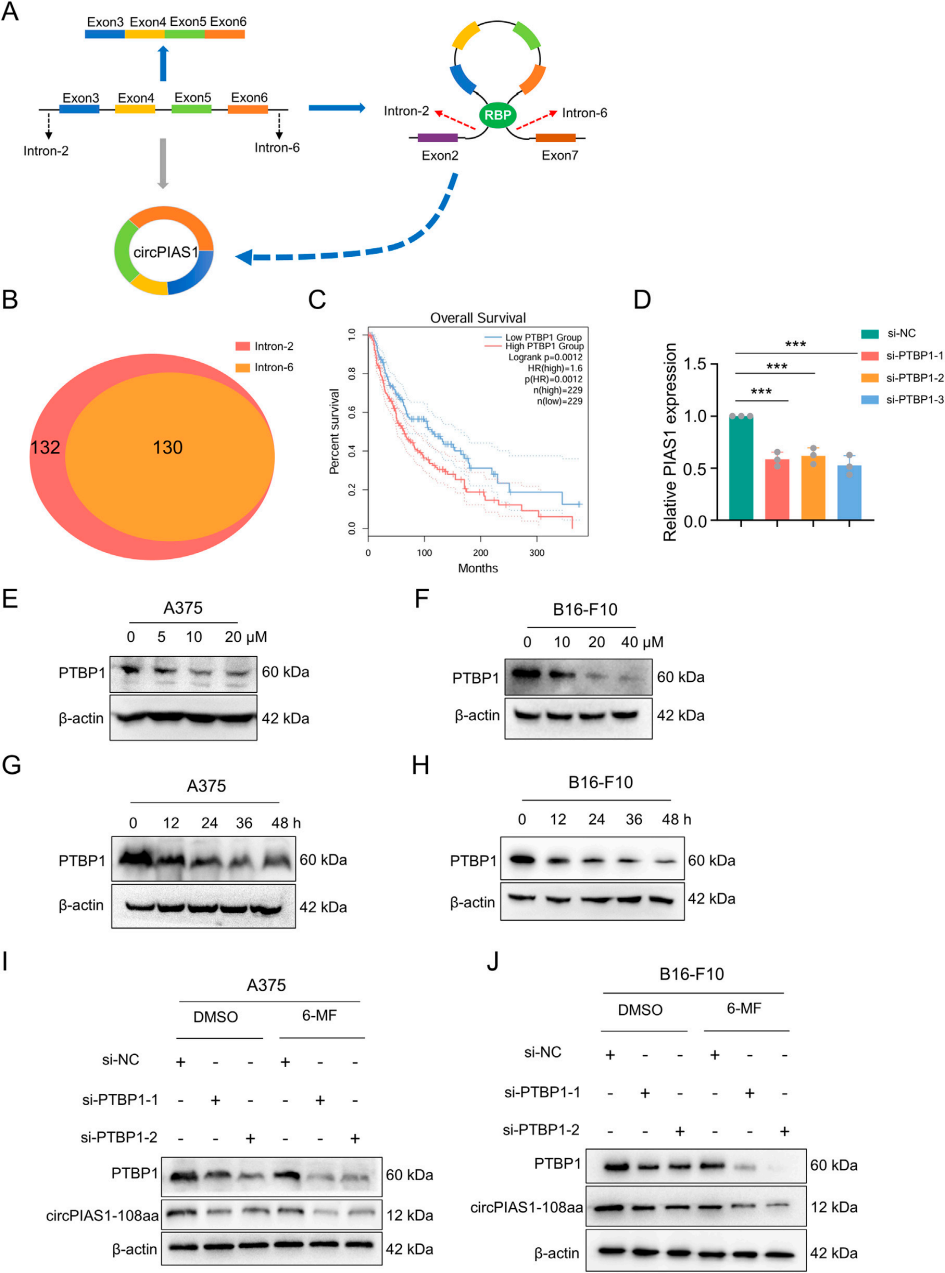
Six-MF blocks circPIAS1 biogenesis by reducing the expression levels of PTBP1. **(A)** CircPIAS1 is generated by the reverse splicing of *PIAS1* exons 3 to 6. RBPs help to bind together introns 2 and 6, and promote the loop formation of exons 3 to 6. **(B)** Using RBPmap database, 130 RBPs were predicted to bind to both intron 2 and intron 6 of the *PIAS1* parent gene. **(C)** The melanoma patients with higher PTBP1 expression had a shorter overall survival (OS) compared to patients with lower PTBP1. **(D)** The knockdown efficiency of circPIAS1 in A375 cells transfected with PTBP1-targeting siRNA for 48 h was detected by qRT-PCR (n = 3 independent experiments). **(E,F)** Western blot experiments were performed to investigate the effect of adding different concentrations of 6-MF at 48 h on PTBP1. β-ACTIN was used as an internal control (n = 3 independent experiments). **(G,H)** Western blot experiments were performed to investigate the effect of adding 20 μM 6-MF at different times on PTBP1. β-ACTIN was used as an internal control (n = 3 independent experiments). **(I,J)** Western blot experiments were performed to explore the effects of adding siPTBP1 and adding 6-MF at a concentration of 20 μM on PTBP1 and circPIAS1-108aa in A375 and B16-F10 cells. β-ACTIN was used as an internal control (n = 3 independent experiments). Data represent three experiments and are expressed as the mean ± SD. ns (not significant), ^*^ *P* < 0.05, and ^**^ *P* < 0.01 (significant). Data were analyzed by One-way ANOVA (D) in GraphPad Prism 10.5.0.

The generation of circRNA is closely related to RBP, which plays a crucial role in the process of reverse splicing (Li et al., 2025). During the biogenesis of the circPIAS1, RBP promotes the bringing together of introns 2 and 6 in the host gene, causing reverse splicing between these two introns and ultimately resulting in the biogenesis of the ring-shaped PIAS1. RBPmap database was utilized for RBP screening (Paz et al., 2014), resulting in the identification of 130 RBPs (Figures 4A,B). Following a comparative analysis of the TCGA and GEPIA2 databases, 9 RBPs were identified as meeting the statistical requirements and demonstrating a significant relationship with the generation of circRNA (Supplementary Figure S2G). Among them, PTBP1 is highly expressed in the skin, and its presence is detrimental to patient survival (Figure 4C). Knockdown of *PTBP1* significantly decreased the gene levels of circPIAS1 (Figure 4D). Western blot experiments demonstrated that the levels of PTBP1 were reduced dose- and time-dependently in the treatment of 6-MF (Figures 4E–H; Supplementary Figure S2A–D). PTBP1 downregulation by siRNAs markedly inhibited the expression of PTBP1 and circPIAS1-108aa. This inhibitory effect was further enhanced when 6-MF was added (Figures 4I,J; Supplementary Figure S2E–F).

### 3.5 The natural metabolite 6-MF enhances the therapeutic effect of PD-1 inhibitors on melanoma

**FIGURE 5:**
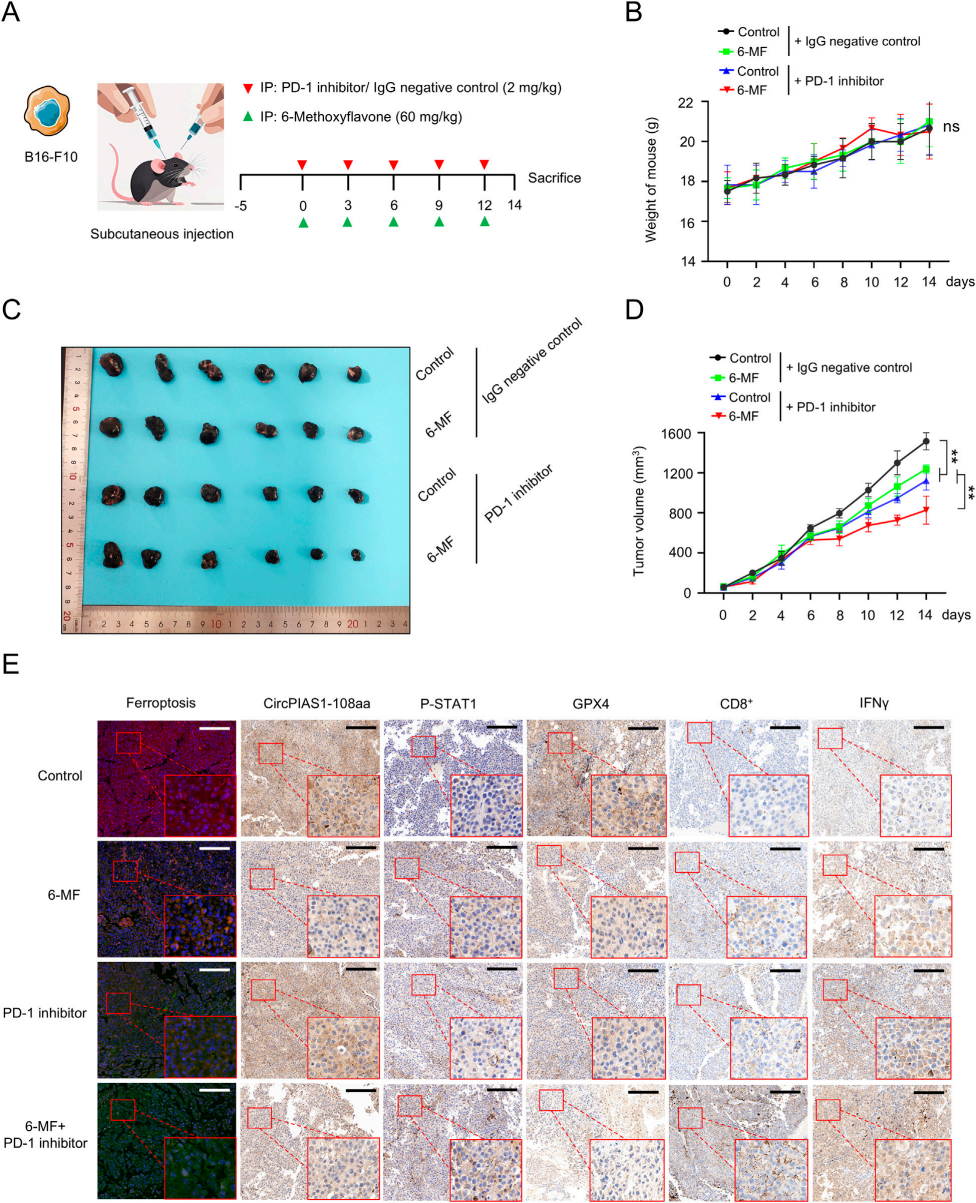
The natural metabolite 6-MF enhances the therapeutic effect of PD-1 inhibitors on melanoma. **(A)** Melanoma model was created by subcutaneously inoculating B16-F10 cells into C57 BL/6J mice. The mice were divided into four groups when the tumors formed. The groups were as follows: negative control; 6-MF; PD-1 inhibitor; and their combination. The mice were administered the treatment every 3 days for five times, starting from day 0. (6-MF was used at a concentration of 60 mg/kg, and PD-1 inhibitor and IgG negative control at 2 mg/kg). **(B)** Changes in the body weight of mice in the different treatment groups. The weight was measured every 2 days (n = 6 per group). **(C)** Melanoma tissues of mice in different treatment groups: negative control; 6-MF; PD-1 inhibitor; and their combination (n = 6 per group). **(D)** Changes in melanoma volume over time for mice in different treatment groups (n = 6 per group). **(E)** IHC staining for ferroptosis, circPIAS1-108aa, P-STAT1, GPX4, CD8^+^, and IFN-γ of melanoma tissues to evaluate various markers. (scale bar: 100 μm) (n = 3 independent experiments). Data were analyzed by One-way ANOVA **(B,D)** in GraphPad Prism 10.5.0.

In order to further elucidate the mechanism of action of 6-MF in melanoma and evaluate its potential value as a therapeutic agent, a melanoma subcutaneous inoculation model was constructed using C57 BL/6J mice (Figure 5A). The experimental animals were randomly divided into four groups: 6-MF monotherapy, PD-1 inhibitor monotherapy, 6-MF and PD-1 inhibitor combination, and IgG negative control. The animal experiment demonstrated that throughout the treatment period, the changes of body weight and pathological morphology of various tissues were not obviously influenced by the intervention (Figure 5B; Supplementary Figure S3B). The analysis of tumor growth demonstrated that the utilization of 6-MF or PD-1 inhibitors as monotherapy was capable of inducing varying degrees of tumor volume reduction, while the combination therapy exhibited remarkably enhanced efficacy (Figures 5C,D).

To elucidate the molecular mechanism by which circPIAS1 suppresses immunogenic ferroptosis by inhibiting *STAT1* phosphorylation, we analyzed tumor tissues using IHC staining technique. The results showed that the levels of P-STAT1 were notably upregulated in the 6-MF or the PD-1 inhibitor group compared with the control group. After combined intervention with 6-MF and PD-1 inhibitor, the expression level of P-STAT1 was prominently increased, while the expression of GPX4 showed a reverse regulatory trend (Figure 5E; Supplementary Figure S3A). As the RNA-seq results were correlated with the immune microenvironment (Figures 2A,B), the present study further elucidated the ferroptotic process and immune microenvironmental characteristics in tumor tissues. The results showed that the 6-MF or PD-1 inhibitor single administration could induce ferroptosis in cancer cells, and promote an increase in the number of CD8^+^T cells and the IFN-γ expression in the immune microenvironment. The combination strategy dramatically enhanced the degree of ferroptosis in cancer cells, and facilitated a distinct increase in the number of CD8^+^T cells and IFN-γ levels in the immune microenvironment (Figure 5E; Supplementary Figure S3A). The results indicate that the combination of 6-MF and PD-1 inhibitors can significantly accelerate the efficacy of immunotherapy for melanoma, opening up new potential avenues for the clinical treatment of melanoma.

## 4 Discussion

In the preceding decade, the advent of ICB has precipitated a paradigm shift in the management of metastatic melanoma, resulting in enhanced patient survival rates. Nevertheless, it should be noted that not all patients respond to ICBs, and there is heterogeneity in tumor response and host toxicity among patients receiving ICBs. Even for cancer types that show high response to ICBs, such as melanoma, a significant proportion of patients’ tumors are either inherently resistant or eventually develop resistance to treatment. This has prompted a need for further investigation into the mechanisms by which host factors within tumors influence the therapeutic efficacy of ICB (Bu et al., 2022; Robinson et al., 2022).

In previous studies, we found that circPIAS1 can act as an oncogene, encoding circPIAS-108aa to promote cancer development. circPIAS1 can inhibit STAT1 phosphorylation, reactivating downstream SLC7A11/GPX4 pathway protein levels (Zang et al., 2024). Subsequent large-scale screening revealed that 6-MF can effectively knock down circPIAS1 expression without affecting the levels of *PIAS1*. The biogenesis of circRNA is dependent on RBP-driven circularization, and numerous RBPs have been demonstrated to bind to introns on both sides of the exon of circRNA to regulate circRNA expression levels (Cui et al., 2022; Yang et al., 2022). Subsequent screening efforts led to the identification of PTBP1 as the RBP of circPIAS1. Further experiments revealed that 6-MF can reduce the protein level of PTBP1, suggesting a potential mechanism through which 6-MF may inhibit the production of circPIAS1 and expression of circPIAS1-108aa by decreasing PTBP1 protein levels. Interestingly, 6-MF did not directly induce ferroptosis, rather, it indirectly regulated the process by inhibiting the *PI3K-AKT* signaling pathway. When combined with IFN-γ at the cellular level or PD-1 inhibitors at the animal level, 6-MF expedited *STAT1* phosphorylation and markedly activated the ferroptosis, resulting in oxidative ferroptosis in melanoma cells. In addition, it has been demonstrated that this remodeling of the immune microenvironment is associated with an enhancement in the efficacy of PD-1 inhibitors, as indicated by an increase in CD8^+^ T cell infiltration. Neither IFN-γ treatment *in vitro* nor PD-1 inhibitor intervention *in vivo* downregulated circPIAS1. This suggests circPIAS1’s high level resists immunotherapy, driving immune evasion and resistance in melanoma.

The dual mechanisms of “targeting the intrinsic pathway of tumor cells and activating the immune response” provide a theoretical foundation for the combined application of flavonoids with immunotherapy. This study establishes a novel link between 6-MF and circRNA-mediated tumor resistance, and provides a theoretical basis for the use of flavonoid combinations in cancer immunotherapy. The natural metabolite 6-MF has demonstrated efficacy *in vitro* and *in vivo*, suggesting potential for use as a therapeutic agent. Its wide range of sources and relatively low toxicity make it a promising candidate for development, with the potential to become a new type of treatment or adjuvant therapy, particularly for patients with drug resistance. The combination strategy may become an important way to improve the treatment effectiveness of melanoma. The flavonoid 6-MF has shown positive prospects in melanoma treatment, which will bring more therapeutic options and hope to melanoma patients.

## 5 Conclusion

In summary, a natural product 6-MF, was identified by library screening. We found that 6-MF inhibited the *PI3K-AKT* signaling pathway and PTBP1 levels, consequently suppressing circPIAS1 biogenesis and circPIAS1-108aa expression from the source. The combination of 6-MF and immunotherapy reduced melanoma proliferative capacity, increased intracellular oxidative stress, and promoted, phosphorylation to inhibit the *SLC7A11/GPX4* signaling pathway and promote ferroptosis in melanoma cells. These findings provide new perspectives for a deeper understanding of the antitumor effects of 6-MF, as well as potential drug targets and therapeutic strategies for the treatment of melanoma.

## Data Availability

The data presented in the study are deposited in the repository Genome Sequence Archive (Genomics, Proteomics & Bioinformatics 2025) in National Genomics Data Center (Nucleic Acids Res 2022), China National Center for Bioinformation / Beijing Institute of Genomics, Chinese Academy of Science, accession number PRJCA047102 https://ngdc.cncb.ac.cn/bioproject/browse/PRJCA047102.

## Abbreviations

circRNA, Circular RNA; 6-MF, 6-methoxyflavone; IFN-γ, Interferon-γ; PD-1 inhibitors, programmed death-1; ICB, immune checkpoint blockade; RNA-seq, Transcriptome sequence; qRT-PCR, Real-time quantitative PCR; EdU, 5-Ethynyl-2′-dexyuridine; CCK-8, Cell counting Kit-8; RBP, RNA binding protein; PTBP1, Polypyrimidine Tract Binding Protein 1.
