# Supplementary material for: Six-methoxyflavone suppresses CircPIAS1 biogenesis via targeting PTBP1 and, in combination with IFN-γ, promotes ferroptosis in melanoma
Source: Front Pharmacol. 2025 Nov 19;16:1681890. doi: 10.3389/fphar.2025.1681890 (PMC12672503; doi:10.3389/fphar.2025.1681890)

Supplementary Material

# Supplementary Figures and Tables

## Supplementary Figures

##
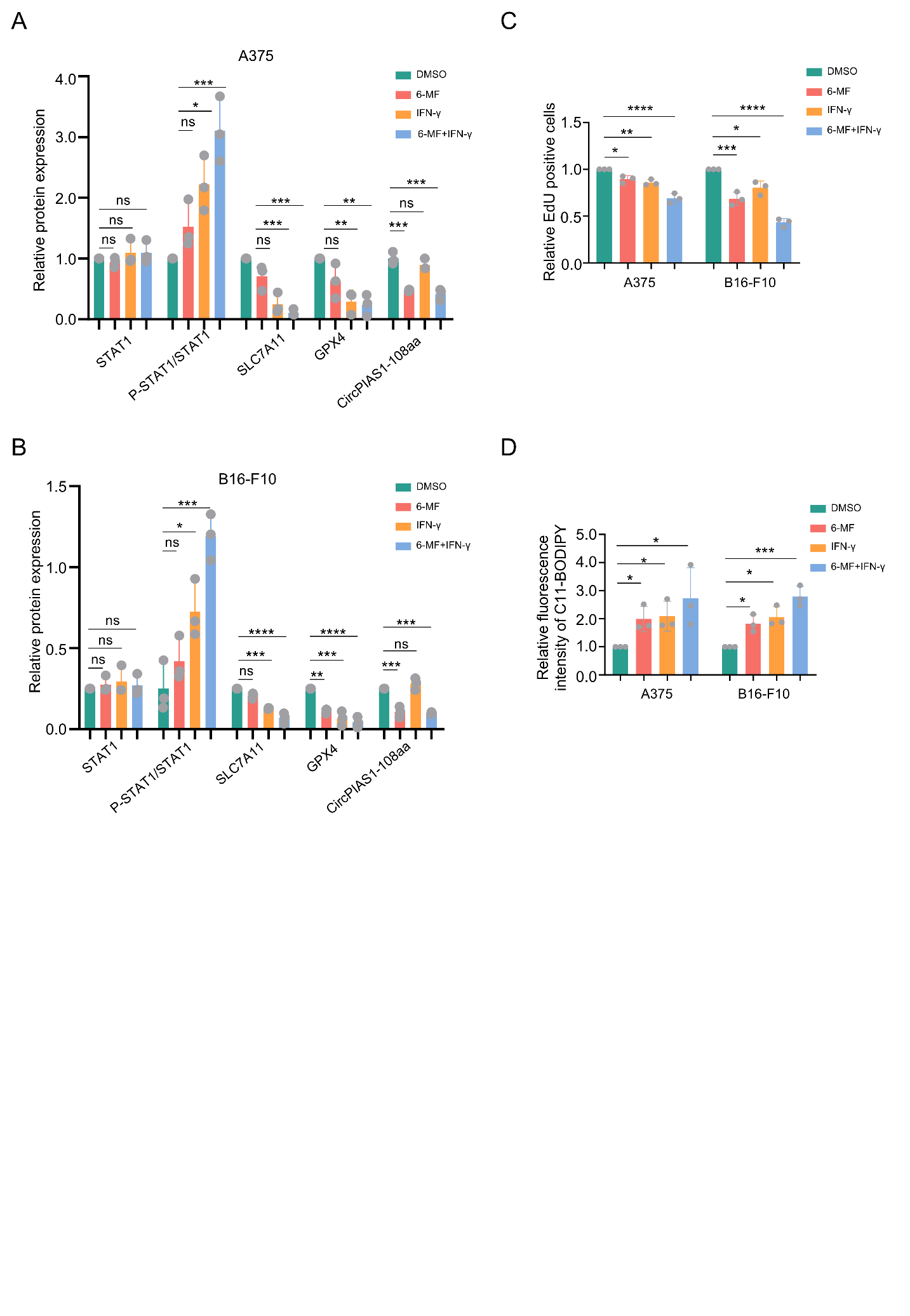


**Supplementary Figure 1 The combination of 6-MF and IFN-γ inhibits the SLC7A11/GPX4 axis via increasing STAT1 phosphorylation and promotes ferroptosis in melanoma cells. A-B.** Effect of 6-MF, IFN-γ and co-administration of 6-MF and IFN-γ on relative protein levels of P-STAT1, STAT1, SLC7A11, GPX4 and circPIAS1-108aa (6-MF was used at a concentration of 20 μM, and IFN-γ at 2 ng/mL) (n = 3 independent experiments). **C.** EdU assay to detect the alteration of cancer cell proliferation by 6-MF, IFN-γ and co-administration of 6-MF and IFN-γ (6-MF was used at a concentration of 20 μM, and IFN-γ at 2 ng/mL.) (n = 3 independent experiments). **D.** Effect of 6-MF, IFN-γ and co-administration of 6-MF and IFN-γ on lipid peroxidation damage on the membrane surface of melanoma cells (6-MF was used at a concentration of 20 μM, and IFN-γ at 2 ng/mL for 48 h.) (n = 3 independent experiments). Data represent three experiments and are expressed as mean ± SD. ns (not significant), **P* < 0.05 and ***P* < 0.01 (significant). Data were analyzed by One-way ANOVA (A, B, C, D) in GraphPad Prism 10.5.0.

**
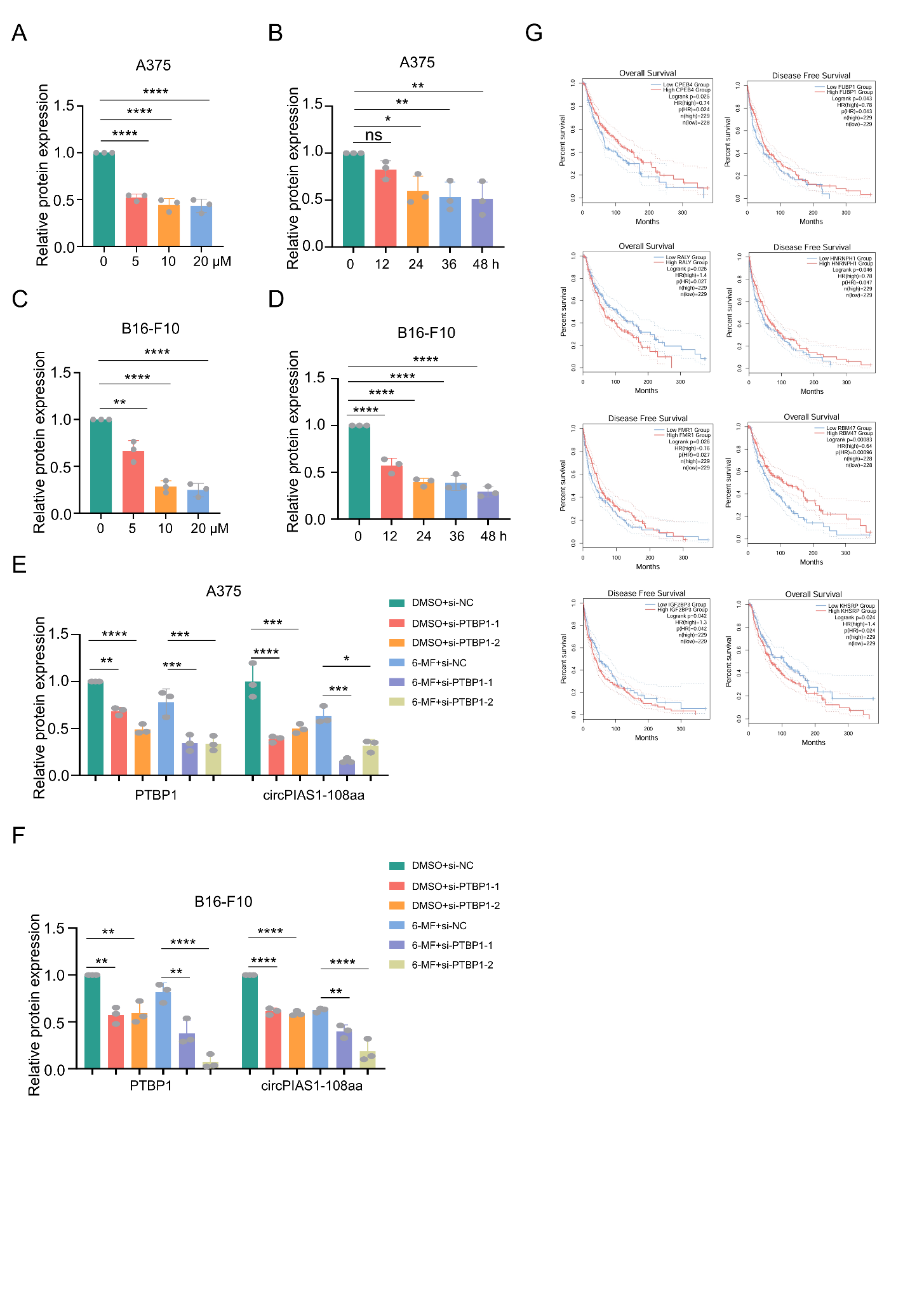
**

**Supplementary Figure 2 Six-MF blocks circPIAS1 biogenesis by reducing the expression levels of PTBP1. A-B.** Relative protein levels of PTBP1, 6-MF added to A375 cells at different concentrations and times (The concentration of 6-MF was 20 μM) (n = 3 independent experiments). **C-D.** Relative protein levels of PTBP1 in B16-F10 cells added with different concentrations and times of 6-MF (The concentration of 6-MF was 20 μM) (n = 3 independent experiments). **E.** Relative protein levels of PTBP1 and circPIAS1-108aa ,6-MF and siPTBP1 added to A375 cells (The concentration of 6-MF was 20 μM.) (n = 3 independent experiments). **F.** Relative protein levels of PTBP1 and circPIAS1-108aa,6-MF and siPTBP1 added to B16-F10 cells (The concentration of 6-MF was 20 μM) (n = 3 independent experiments). **G.** Overall survival and disease free survival analysis of melanoma patients in the GEPIA2 database. Data represent three experiments and are expressed as mean ± SD. ns (not significant), **P*<0.05 and ***P*<0.01 (significant). Data were analyzed by two-tailed Student's t-tests (F) and One-way ANOVA (A, B, C, D, E, F) in GraphPad Prism 10.5.0.


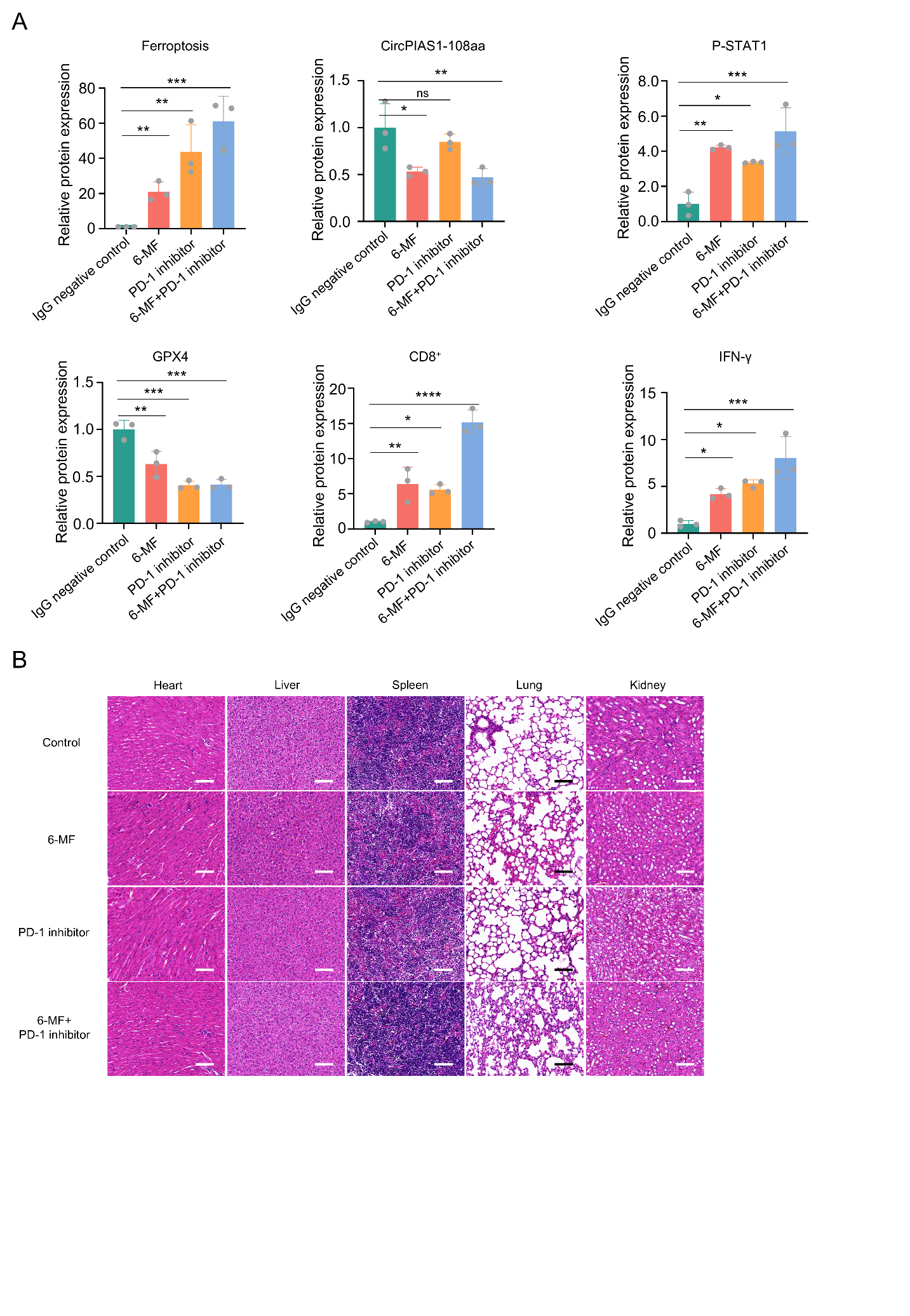


**Supplementary Figure 3 The natural compound 6-MF enhances the therapeutic effect of PD-1 inhibitors on melanoma. A.** Effects of different treatment groups on various key markers (ferroptosis, circPIAS1-108aa, P-STAT1, GPX4, CD8^+^, IFN-γ) in mouse melanoma tissue (n = 3 independent experiments). **B.** Analysis of the effects of different treatment groups on the hearts, livers, spleens, lungs and kidneys of mice. (scale bar: 100 μm) (n = 3 independent experiments). Data represent three experiments and are expressed as mean ± SD. ns (not significant), **P* < 0.05, and ***P* < 0.01 (significant). Data were analyzed by One-way ANOVA (A) in GraphPad Prism 10.5.0.

## Supplementary Tables

**Supplementary Table 1. The qRT-PCR primers sequences**

| **Genes** | **Primers** | **Sequences** |  |
| --- | --- | --- | --- |
| *hsa_circ_0008378*  (divergent primer) | Forward | 5'-CGACCCAGCCGACCAATTA-3' | |
|  | Reverse | 5'-GACTGTTGTCTGATGTCTTCCAA-3' | |
| *H-PIAS1* | Forward | 5'-CAATGGAAGCAGTAGTGGCAGTAAC-3' | |
|  | Reverse | 5'-TTGTGACGGTGTGGCTATGGC-3' | |
| *H-GAPDH* | Forward | 5'-GGTGAAGGTCGGAGTCAACGG-3' | |
|  | Reverse | 5'-GAGGTCAATGAAGGGGTCATTG-3' | |

**Supplementary Table 2. The antibody information**

| **Target protein** | **Source** | **Catalog Number** | **Application** |
| --- | --- | --- | --- |
| PTBP1 | Proteintech | 12582-1-AP | WB |
| STAT1 | CST | 14994S | WB |
| P-STAT1 (Tyr701) | CST | 9167S | WB/IHC |
| SLC7A11 | HUABIO | HA721868 | WB |
| GPX4 | Proteintech | 67763-1-Ig | WB/IHC |
| β-ACTIN | Proteintech | 66009-1-Ig | WB |
| AKT | Proteintech | 10176-2-AP | WB |
| P-AKT | Proteintech | 66444-1-Ig | WB |
| PI3K | Proteintech | 60225-1-Ig | WB |
| P-PI3K | Abcam | ab127617 | WB |
| CD8^+^ T | Servicebio | GB12068 | IHC |
| IFNγ | AiFang Biological | AF02446 | IHC |

**Supplementary Table 3. The sequences of siRNAs**

| **Target genes** | **Type** | **Target sequence** |
| --- | --- | --- |
| *H-PTBP1* | siRNA | #1:5'-GCUUCUGCAGCAAACGGAA-3' |
|  | siRNA | #2:5'-GGCACAGUGUUGAAGAUCA-3' |
|  | siRNA | #3:5'-CCAACACCAUGGUGAACUA-3' |
| *M-PTBP1* | siRNA | #1:5'-CCAACACUAUGGUUAACUA-3' |
|  | siRNA | #2:5'-GAGCAGAGACUACACUCGA-3' |

**Report on preparation of polyclonal antibodies**


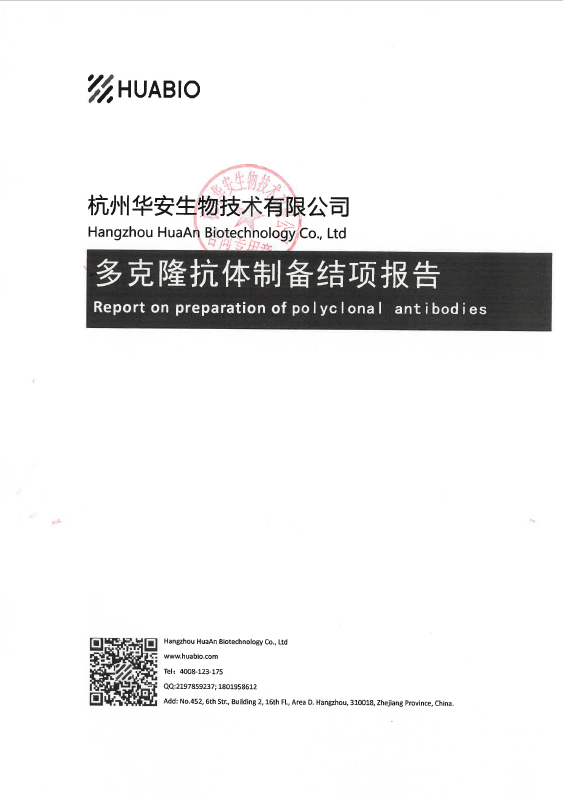


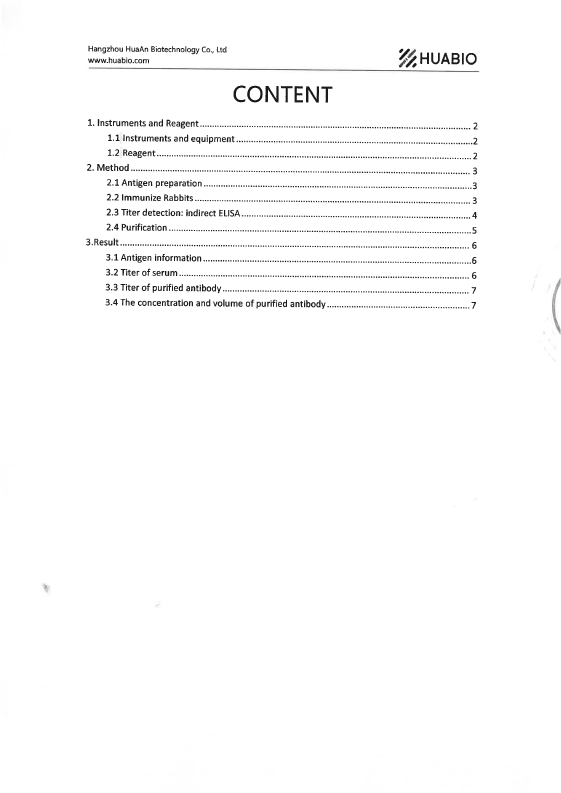

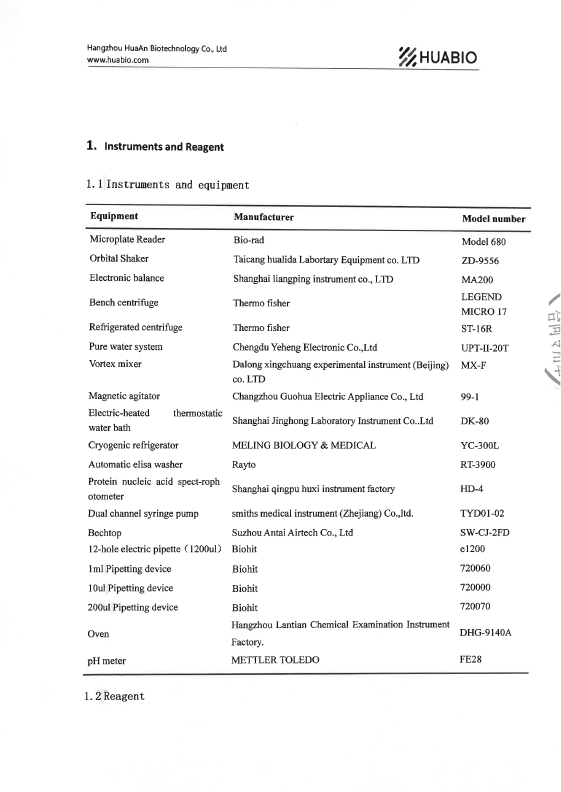


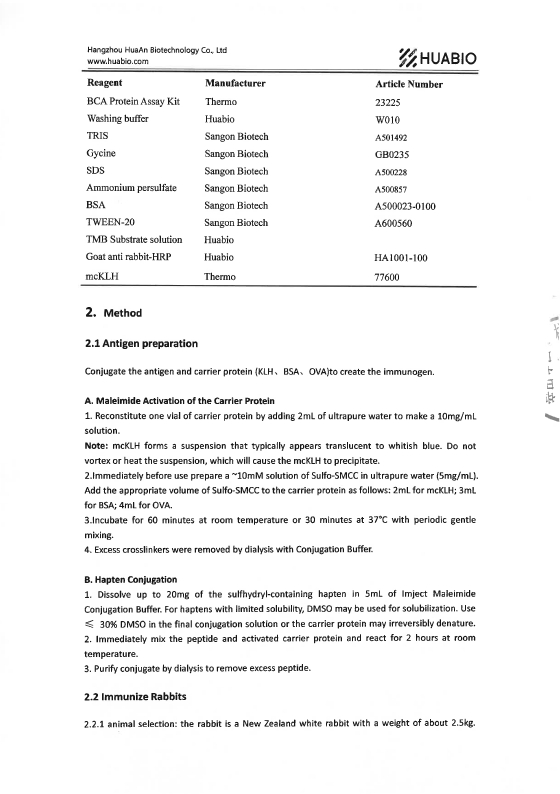


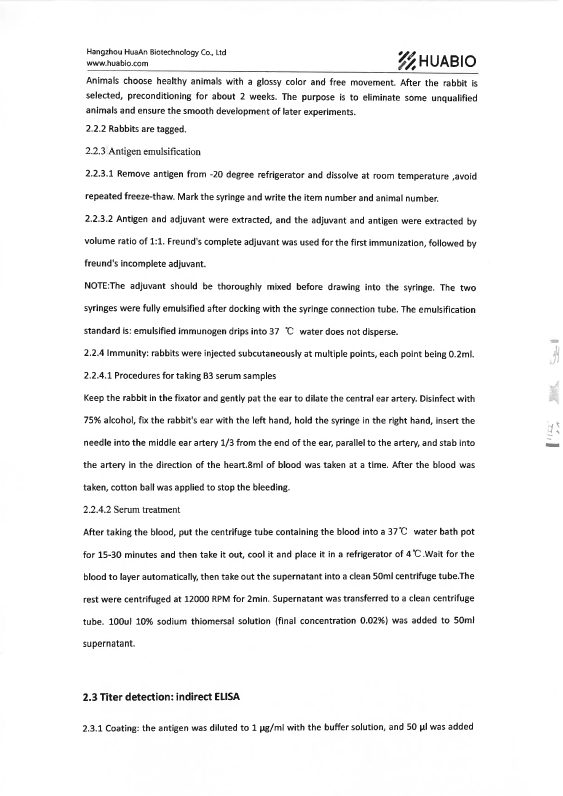


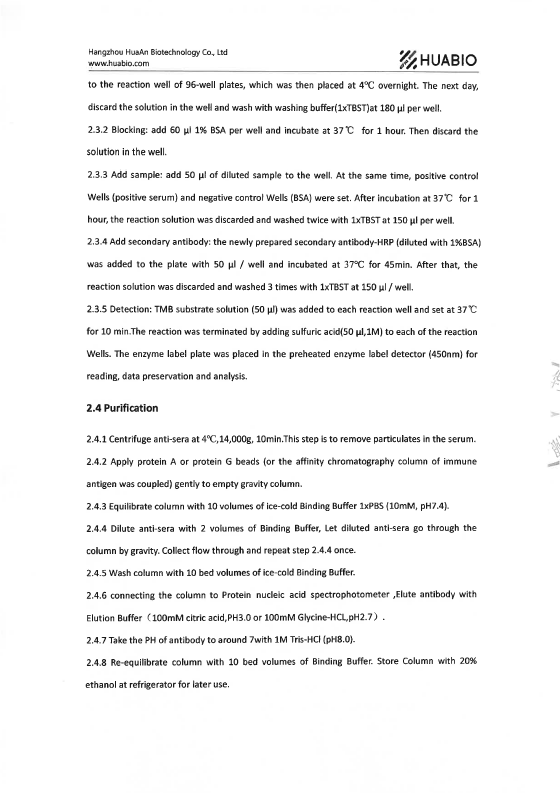


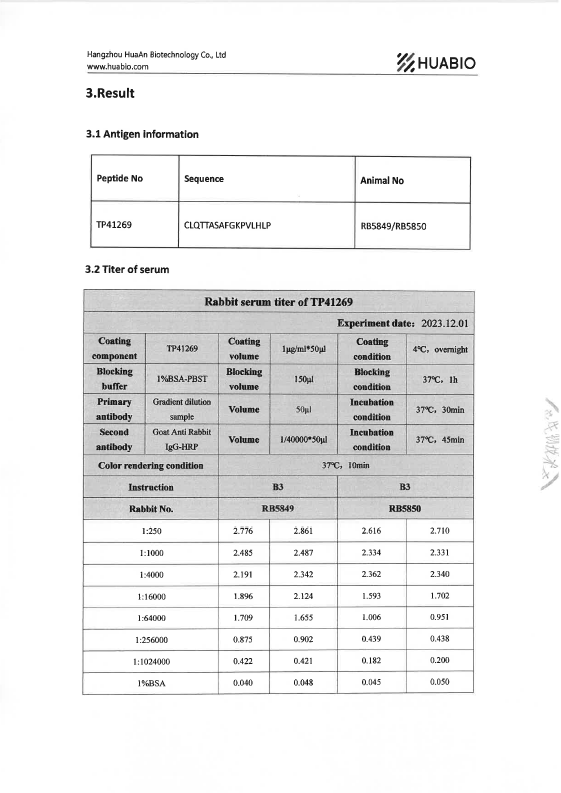


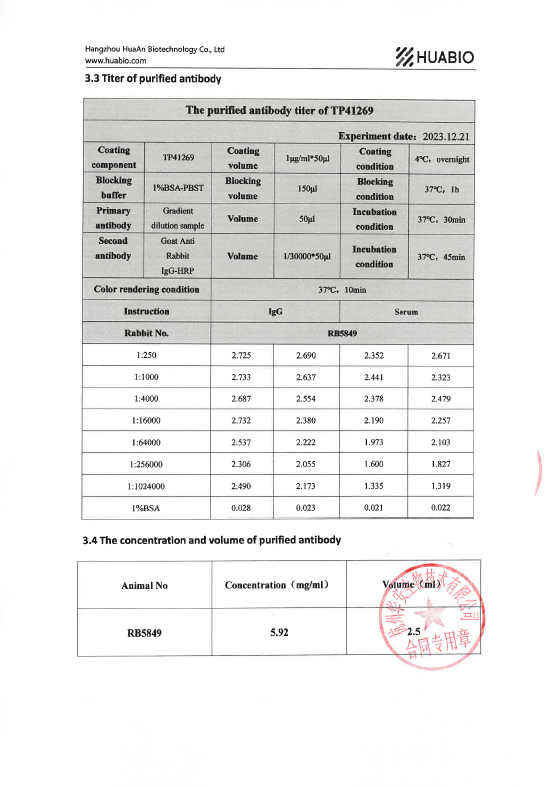

Supplement: Supplementary file 1 [file Supplementaryfile1.docx]
